# Expression of MicroRNAs in Periodontal Disease: A Systematic Review

**DOI:** 10.1155/2021/2069410

**Published:** 2021-01-19

**Authors:** María Verónica Cuevas-González, Fernando Suaste-Olmos, Alma Graciela García-Calderón, Karla Lizette Tovar-Carrillo, León Francisco Espinosa-Cristóbal, Salvador David Nava-Martínez, Juan Carlos Cuevas-González, Graciela Zambrano-Galván, Rosa Alicia Saucedo-Acuña, Alejandro Donohue-Cornejo

**Affiliations:** ^1^School of Dentistry, National Autonomous University of Mexico, Mexico City, Mexico; ^2^Institute of Cellular Physiology, National Autonomous University of Mexico, Mexico City, Mexico; ^3^Department of Stomatology, Institute of Biomedical Sciences, Autonomous University of Ciudad Juárez, Ciudad Juárez, Chihuahua, Mexico; ^4^School of Dentistry, Juarez University of the State of Durango, Durango, Dgo, Mexico; ^5^Department of Biological and Chemical Sciences, Institute of Biomedical Sciences, Autonomous University of Ciudad Juárez, Ciudad Juárez, Chihuahua, Mexico

## Abstract

**Introduction:**

Periodontal disease (PD) is a chronic inflammation of the soft tissues that support the structure of the tooth, and miRNAs are highly dynamic molecules that participate in the regulation of gene expression interfering with multiple genetic targets. The dysregulation of the expression of miRNAs has been associated with different types of pathologies; therefore, they are excellent molecules to be studied as biomarkers. *Material and Methods*. A search was made in the electronic databases of PubMed, Scopus, and Science Direct. The following key words were used: “microRNAs,” “miRNAs,” “periodontal disease,” “periodontitis,” and “biomarker”; employee independent search strategies with the Boolean operators “OR” and “AND”; a further search of the references of the selected studies was performed to detect potential studies that met the selection criteria. The data recollected from each article were author, country, year of publication, sample size, type of sample used to identify miRNAs, methodology used to identify miRNAs, type of periodontal disease, and miRNAs identified.

**Results:**

Of the 13 selected studies, 6 used gingival tissue as a sample for the identification of miRNAs, 3 used gingival fluid, 2 used saliva, 1 used serum, and another used periodontal tissue. Chronic periodontitis was the most studied periodontal disease in 9 of the 13 selected articles; 7 used microarrays as the main technique for the identification of miRNAs. qRT-PCR was the assay choice to validate the identified miRNAs.

**Conclusion:**

The main type of periodontal disease on which most studies are focused is chronic periodontitis, with the main miRNAs being hsa-miR-146a, hsa-miR-146b, hsa-miR-155, and hsa-miR-200. This systematic review is one of the first to carry out an analysis of the current role of miRNAs in PD as biomarkers.

## 1. Introduction

Periodontal disease (PD) is a chronic inflammation of the soft and hard tissues that support the structure of the tooth. From 10% to 90% of the world population has some type of alteration at this level [1]; however, these data change considerably according to region. For example, in developing countries, between 36% and 64% of adults have dental calculus compared with developed countries (between 14% and 47%) [2]. These data show lack of health care orally and lack of early diagnosis—both of which would prevent the development of PD.

Clinical parameters are used to assess PD, clinical attachment loss, probing depth, the presence and extent of angular bony defects, and furcation involvement, among others, go hand in hand with imaging tests, such as radiographs or tomography, to aid in the assessment of bone loss [3]; these parameters help to determine the treatment and the prognosis of the pathology. Therefore, its usefulness becomes limited when establishing an early diagnosis. Hence, there is a need to identify biomarkers that support the early diagnosis of PD in order to decrease its incidence.

Identifying molecules that allow the diagnosis, progression, or response to pharmacological treatments for different conditions—particularly PD through a noninvasive approach—has meant that miRNAs are excellent candidates to determine the different stages of this condition [4, 5]. miRNAs are highly dynamic molecules that participate in the regulation of gene expression interfering with multiple genetic targets. This is done by inhibiting the transcription and translation of a gene or associating with chromatin remodeling proteins, which silence or activate the expression of multiple genes [6, 7]. Furthermore, miRNAs get out of the cell through small vesicles named exosomes and, through this mechanism, they control different targets remotely [8]. Unlike proteins or mRNA (messenger RNA), miRNAs have been shown to be highly stable in different types of biological samples and are able to be isolated from tissues and biofluids or from samples embedded in paraffin [9].

The dysregulation of the expression of miRNAs has been associated with different types of pathologies; therefore, they are excellent molecules to be studied as biomarkers [9]. However, miRNAs face different methodological limitations derived from their own biology. Multiple research groups have focused on the identification of miRNAs in different types of PD using different methodologies and different types of samples, so the objective of this work was to carry out a systematic review focused on identifying the main miRNAs related to PD as well as defining the methodology used for such identification and the challenges that need to be overcome as it is studied.

To carry out this work, original articles of the case and control type or comparative descriptions that evaluated the expression of miRNAs in subjects with periodontal disease confirmed by clinical and/or imaging criteria were reviewed.

## 2. Material and Methods

### 2.1. Research Question

What are the microRNAs proposed as possible biomarkers for identification of different types of PD in humans?

### 2.2. Inclusion Criteria

- Comparative descriptive or case-control or descriptive design studies
- Studies that will have a clinical and/or imaging diagnosis that will confirm the presence of PD
- Studies that will take into account the classification of PE from 1999 or 2017
- Studies that relate the presence of PD to the expression of miRNAs
- Studies whose methodology will explicitly explain the miRNA identification mechanism

### 2.3. Exclusion Criteria

In vitro studies, literature reviews, systematic reviews or meta-analysis, and letters to the editor, as well as studies in a language other than English or Spanish, were excluded.

### 2.4. Strategy Research

A search was made in the electronic databases of PubMed, Scopus, and Science Direct. The revision was carried out from 2010 to 2020, and the following key words were used: “microRNAs,” “miRNAs,” “periodontal disease,” “periodontitis,” and “biomarker,”; employee independent search strategies with the Boolean operators “OR” and “AND”; a further search of the references of the selected studies was performed to detect potential studies that met the selection criteria (PROSPERO registration number CRD42021222743).

### 2.5. Study Selection

For study selection, an initial filter was performed by title and summary, which mentioned the study of miRNAs or microRNAs in periodontal disease. The selected studies were placed in a database in which a second full-text filter was performed identifying a specific bibliography that met the established selection criteria. The selection of the studies was carried out independently by two examiners; in case of discrepancy, a third evaluator participated.

### 2.6. Extraction and Data Analysis

The data recollected from each article were author, country, year of publication, sample size, type of sample used to identify miRNAs, methodology used to identify miRNAs, type of periodontal disease, and miRNAs identified; to do this, the miRNAs reported by the selected articles according to the selection criteria were placed in a database and descriptive statistics were made based on frequencies. Finally, if they were validated, independently, data related to the processing methodology were collected, such as the nucleic acid extraction method, the type of assay used to identify miRNAs, and the type of normalization control. Descriptive statistics of the data were performed using the SPSS V.22 program.

### 2.7. Risk Bias

To determine the risk of bias, 1 (QUADAS-2) was used. This tool comprises four domains: patient selection, index test, reference standard, and flow and timing. This assessment was carried out by two examiners independently.

## 3. Results

### 3.1. Research Strategy

**Figure 1:**
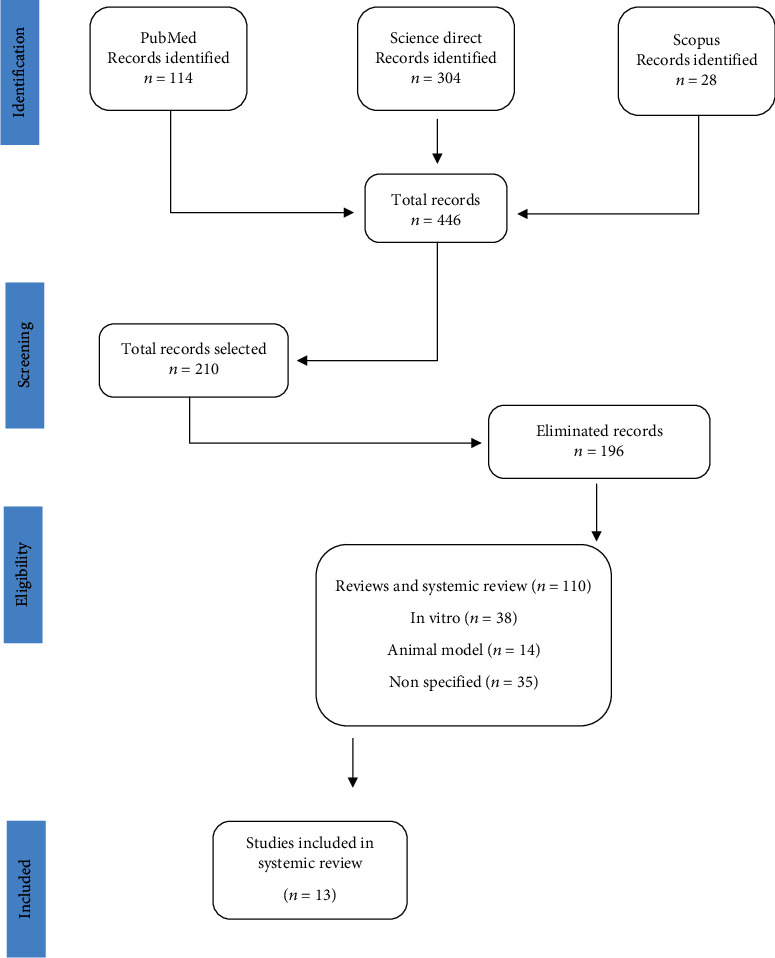
Search strategy.

In the first search, 446 studies were identified; in the second phase of filtering, which was performed based on the established selection criteria, only 13 studies were selected (Figure 1). A total of 433 papers were deleted (literature reviews, systematic reviews, and animal or in vitro studies).

### 3.2. Study Characteristics

### 3.3. Risk of Bias

After applying QUADAS-2, it was observed that 100% of the included studies had all the elements required for diagnostic studies and were therefore classified as having a low risk of bias (Table 1).

### 3.4. Data Description

Of the 13 selected studies, 6 used gingival tissue as a sample for the identification of miRNAs, 3 used gingival fluid, 2 used saliva, 1 used serum, and another used periodontal tissue. Chronic periodontitis was the most studied periodontal disease in 9 of the 13 selected articles; 7 used microarrays as the main technique for the identification of miRNAs. qRT-PCR was the assay choice to validate the identified miRNAs (Supplementary (available here)) [10–21].

When analyzing the miRNAs proposed in the different included studies, according to the statistical frequency, it was observed that the information is very heterogeneous. However, among these studies, the most reported were hsa-miR-146a and hsa-miR-146b and hsa-miR-155 and hsa-miR-200 related to chronic periodontitis mainly.

## 4. Discussion

The development of PD is a complex multifactorial process: bacterial colonization protected by a matrix formed by polysaccharides and glycoproteins triggers the host's immune response and generates a sustained inflammatory process [22]. However, not all types of PD generate the classic signs of inflammation, as is the case of noninflammatory destructive periodontal disease. This is characterized by loss of periodontal ligament union, bone loss, and generalized gingival recession without the patient having any signs of gingival inflammation [23]. This complicates the early diagnosis and favors the destruction of periodontal tissue (resulting in teeth loss), so a large part of research in the field of periodontics has focused on the identification of biomarkers that will help with the early diagnosis of PD.

The first step in the study of biomarkers is to determine the type of sample to be used, as its specificity will depend on this. Without doubt, we can consider fresh tissue as a gold standard in the identification of future biomolecules that determine the presence or absence of any pathology or its future behavior. However, the availability of fresh tissue is often conditioned by its location, the patient's medical condition, the rapid development of the disease, or by the proposed research study design, which makes paraffin-embedded tissues the most viable option conditioned by well-standardized processing techniques. In the literature, there are discrepancies about the quality of genetic material from this type of sample. RNA tends to degrade not only due to enzymatic factors but also due to the chemical reagents used during its processing (e.g., methylol), which prevents the synthesis of cDNA (complementary DNA), or during the process fixation of the tissue. The long RNA chains are more susceptible to generate cross-links with proteins, which makes their isolation and identification complicated. However, the opposite has been shown: the study of small RNAs in paraffin-embedded tissue is viable, maintaining sufficient integrity and quality for their study [24, 25].

One of the main objectives of a biomarker is its easy identification; thus, the use of tissue is often limited to the identification phase. In the clinic, the reason why the biofluids are more viable is because their availability is less invasive for the patient. As already mentioned, the cell releases miRNAs into the circulation in two different ways: either linked to a protein complex (high-density lipoprotein, Argonaute 2 and nucleophosmin-1), which protects them from the enzymes present into the biofluids, or inside microvesicles such as exosomes. Therefore, the quality of miRNAs is not diminished, which makes biofluids a reliable source. It should be emphasized that identifying a specific panel of miRNAs related to a certain pathology from a biofluid is not specific if the tissue has not been studied previously in tissue and biofluid at the same time [26]. In this review, according to the results obtained, 7 of the 13 included studies employed periodontal tissue as a sample to identify miRNAs; the rest of the studies used biofluids, such as crevicular fluid, saliva, and serum—some of which had performed a previous bioinformatic analysis in databases where miRNAs were identified in tissue.

The expression of a specific miRNA can vary considerably from one tissue to another; its unique structural characteristics represent a challenge for the study of expression profiles when routine genetic analysis techniques (e.g., RT-PCR or qRT-PCR) are used. These structural characteristics include the following: (a) its size of 22 nt, which represents an impediment to the use conventional primers; (b) do not have a consensus sequence (e.g., poly-A ends) that allows its retro transcription; (c) the fact that miRNAs within a family can vary by only one nucleotide; and (d) the GC (guanine-cytosine nucleotides) content of each miRNA generates a wide range of melting temperatures, which can lead to a bias towards a single type of miRNA when analyzed in bulk [27–29].

The role of miRNAs in the control of cellular homeostasis positions them as excellent targets for study in the clinical area; hence, analyzing a set of miRNAs associated with a certain biological function or a large group of miRNAs with heterogeneous functions is an experimental strategy that represents a great challenge. For the study of a large number of miRNAs simultaneously, three main types of assays have been developed: qRT-PCR, the hybridization method (microarrays), and massive sequencing (RNA-seq). Each of these experimental strategies offers different kinds of results according to their scope. However, the success of this type of methodology varies according to its work scheme, which generally includes fundamental steps such as obtaining and storing the sample to be analyzed, the extraction of the RNA, and the determination of its quantity and quality [29]. This systematic review gives us a clear picture of the challenge behind the study of miRNAs as biomarkers, as well as the great need to unify the sample processing criteria in order to make a clear comparison of the results between different research groups.

qRT-PCR is a tool that offers high sensitivity and specificity in the detection and quantification of miRNA species. This is because the use of a stem-loop oligonucleotide has been designed to perform the specific reverse transcription of the miRNA of interest followed by a PCR reaction using a TaqMan probe. However, the limitation of this strategy is the analysis of miRNAs already reported and not the de novo search [30]. Hybridization methods, such as microarrays, offer the ability to analyze a large number of targets (e.g., qRT-PCR); they are not employed for the detection of new forms of miRNAs. The limitations of this methodology are as follows: (a) the Tm (melting temperature) of each miRNA can vary considerably; (b) therefore, the different platforms that exist for this type of analysis give different results, making it difficult to compare results with other research groups; and (c) finally, the massive sequencing method (RNA-seq) is highly sensitive in identifying miRNAs in a biological sample, but it is unable to determine an absolute quantification [29].

According to the results obtained, the miRNAs proposed as future biomarkers for PD were heterogeneous. Among the most relevant is hsa-miR-21-3p, which is related to the cancer signaling pathway MAPK, T cell receptors, adhesion molecules, etc. [31]. hsa-miR-146 in conjunction with miR-155 is related to the key regulators of the immune system by inducing the expression of certain cytokines, such as tumor necrosis factor alpha alfa (TNF*α*), IL-1*β*, type I and type II, interferons (IFNs), or RANKL; hence, the expression of both miRNAs is related to any process that involves chronic inflammation [32]. Finally, miR-200 has been related to the mesenchymal epithelial transition by regulating the expression of transcription factor ZEB-1 [33]. Until now, it is clinically complex to determine a panel of miRNAs as biomarkers for PD because the studies carried out are part of the first phase in the study of biomarkers; therefore, it is imperative to validate population studies.

## 5. Conclusion

PD is a complex disease not only because of its large number of etiological factors, but also because of its different classifications. Research studies that have proposed several miRNAs as biomarkers for PD have been based on the 1999 classification; however, in 2017, a new classification involving a large number of PD subtypes was released. Therefore, one of the main challenges is to design validation studies that take into account this new classification in order to determine the specificity and sensitivity of the miRNAs. The main type of PD on which most studies are focused is chronic periodontitis, when analyzing the frequency, the main miRNAs being hsa-miR-146a, hsa-miR-146b, hsa-miR-155, and hsa-miR-200. This systematic review is one of the first to carry out an analysis of the current role of miRNAs in PD as biomarkers. However, although progress has been made in leaps and bounds, more studies are needed to validate what has already been identified.

## Figures and Tables

**Table 1:** Risk of bias of selected studies which shows that 100% of the included studies have a low level of bias.

Author	Year	Patient selection	Index test	Reference standard	Flow and timing	Risk of bias
Yoneda T.	2019	^∗^	^∗^	^∗^	^∗^	Low
Nisha KJ	2019	^∗^	^∗^	^∗^	^∗^	Low
Fujimori K.	2019	^∗^	^∗^	^∗^	^∗^	Low
Jianjia L	2018	^∗^	^∗^	^∗^	^∗^	Low
Micó-Martínez P	2018	^∗^	^∗^	^∗^	^∗^	Low
Amaral SA.	2018	^∗^	^∗^	^∗^	^∗^	Low
Ghotloo S.	2018	^∗^	^∗^	^∗^	^∗^	Low
Radović N.	2018	^∗^	^∗^	^∗^	^∗^	Low
Hee Sam Na	2016	^∗^	^∗^	^∗^	^∗^	Low
Saito A.	2017	^∗^	^∗^	^∗^	^∗^	Low
Motedayyen H.	2015	^∗^	^∗^	^∗^	^∗^	Low
Stoecklin-Wasmer C.	2012	^∗^	^∗^	^∗^	^∗^	Low
Yu-feng Xie	2011	^∗^	^∗^	^∗^	^∗^	Low

## Data Availability

All data obtained from this study can be found in the Institute of Biomedical Sciences of the Autonomous University of Ciudad Juarez, Chihuahua, and can be requested through the corresponding author.

## Supplementary Materials

Supplementary Materials Characteristics of the included studies, the different study variables are described, and the analysis was carried out in two phases: identification phase where data was collected as identification method of miRNAs and RNA isolation method as well as whether the validation is the same. In a second phase, the validation phase was analyzed, where information related to the processing of the samples was collected.
