# Supplementary material for: Expression of MicroRNAs in Periodontal Disease: A Systematic Review
Source: Biomed Res Int. 2021 Jan 19;2021:2069410. doi: 10.1155/2021/2069410 (PMC7840252; doi:10.1155/2021/2069410)
Supplement: Supplementary Materials — Characteristics of the included studies, the different study variables are described, and the analysis was carried out in two phases: identification phase where data was collected as identification method of miRNAs and RNA isolation method as well as whether the validation is the same. In a second phase, the validation phase was analyzed, where information related to the processing of the samples was collected. [file 2069410.f1.pdf]

					IDENTIFICATION PHASE				VALIDATION PHASE						
**Autor**	**Type of sample**	**Sample size**		**Periodontophaty**	**miRNAs Profiling Method**	**RNA Isolation Method**	**miRNA selection fold change >2, p value <0.05**	**Validation**	**TaqMan Micro RNA assay/ qRT-PCR**	**RNA Isolation Method**	**Quality RNA analysis**	**Normalization control**	**Specificity and sensitivity statistics**	**MicroRNAs detectados**	**REF**
		**Cases**	**Controls**												
Yoneda T. (2019)	serum	30	30	Chronic periodontitis	Microarray	3D-Gene® RNA extraction	yes	yes	yes	microRNA Extractor SP kit (Wako, Osaka, Japan)	yes	U6 snRNA	yes	hsa-miR-664a-3p hsa-miR-501-5p hsa-miR-21-3p	(19)
Nisha KJ (2019)	Saliva	32	32	Chronic periodontitis	NGS/RNAseq	TRIzol Reagent (Invitrogen), Thermo Fisher Scientific, Waltham, MA.	yes	yes	N/D	RNAsol Kit, Chromous Biotech Pvt., Bangalore, India.	yes	RNU6-2	N/D	hsa-miR-143-3p	(22)
Fujimori K. (2019)	saliva	120	-	chronic periodontitis	The Inflammatory Response and Autoimmunity miScript miRNAs PCR Array. (SA Biosciences, Frederick, MD)	Total exosome RNA isolation kits (Invitrogen, Carlsbad, CA, USA)	yes	yes	yes	Total exosome RNA isolation kits (Invitrogen, Carlsbad, CA, USA	yes	U6 snRNA	N/D	hsa-381-3p	(23)
Jianjia L (2018)	Gingival tissue	16	16	Chronic periodontitis	Microarray (miRNA profiling in inflamed gingiva from	N/D	N/D	yes	N/D	RNAiso Plus (Takara Bio Inc., Dalian, China)	N/D	U6 snRNA	N/D	miR-144- 5p miR-204	(24)
Micó-Martínez P (2018)	Gingival crevicular fluid	9	9	moderate and severe periodontitis	ND (mirPath v3 KEGG Reverse Search (selecting the method TarBase v7.0)) ND (mirPath v3 KEGG Reverse Search (selecting the method TarBase v7.0))	N/D	N/D	N/D	yes	miRNeasy Serum/ Plasma kit (Qiagen, CA. USA).	N/D	hsa-miR-16-5p (000391	N/D	miR-1226-5p	(25)

Amaral SA. (2018)	Periodontal tissue	18	-	Chronic and aggressive periodontitis	TaqMan® OpenArray® Human MicroRNA Panel, Applied Biosystems	miRNA isolation kit (Ambion mirVana™ miRNA Isolation Kit, Life Technologies, Vilnius, LT, USA)	N/D	N/D	N/D	N/D	N/D		N/D	hsa-miR-1274b, hsa-let-7b-5p, hsa-miR-24-3p, hsa-miR-19b-3p, hsa-miR-720, hsa-miR-126-3p, hsa-miR-17-3p	(26)
Ghotloo S.	Gingival tissue	18	10	Generalized aggressive periodontitis			N/D	N/D	N/D	N/D	N/D		N/D	miR-146a	(27)
Radović N. (2018)	Gingival crevicular fluid	48	48	Chronic periodontitis			N/D	N/D	N/D	N/D	N/D		N/D	miR-146a<br>miR-155	(28)
Hee Sam Na (2016)	Gingival tissue	14	-	Chronic periodontitis	miRNA PCR array. systemRT2 miRNA PCR array system, SABiosciences, Frederick, MD	miRNA isolation kitAmbion, Thermo Fisher Scientific, Waltham, MA.	N/D	N/D	N/D	N/D	N/D	RNU44	N/D	miRNA-15b<br>miRNA-211<br>miRNA-372<br>miRNA-656<br>miRNA-128<br>miRNA-34a<br>miRNA-38	(29)
Saito A. (2017)	Gingival crevicular fluid	11	9	non specify	miRNA PCR panel (Exiqon). miRCURY LNATM Universal RT microRNA PCR System (Exiqon, Vedbæk, Denmark)	Trizol LS reagent (Life Technologies, Carlsbad, CA, USA)	N/D	yes	N/D	N/D	N/D	N/D	N/D	hsa-miR-19a-3p<br>hsa-miR-181b-5p<br>hsa-miR-155-5p<br>hsa-miR-200b-3p<br>hsa-miR-125b-5p<br>hsa-miR-31-3p<br>hsa-miR-100-5p<br>hsa-miR-205-5p<br>hsa-miR-223-3p<br>hsa-miR-200a-5p<br>hsa-let-7e-5p<br>hsa-miR-30e-5p<br>hsa-miR-200c-5p<br>hsa-miR-140-5p<br>hsa-miR-125a-5p<br>hsa-miR-187-3p<br>hsa-miR-338-3p<br>hsa-miR-590-5p<br>hsa-miR-101-3p<br>hsa-miR-200c-3p<br>hsa-miR-21-3p<br>hsa-let-7i-5p<br>hsa-miR-320a<br>hsa-miR-301a-3p<br>hsa-miR-99a-5p<br>hsa-miR-194-5p<br>hsa-miR-145-5p<br>hsa-miR-30e-3p	(30)

														hsa-miR-222-3p<br>hsa-miR-26b-5p<br>hsa-miR-144-3p<br>hsa-miR-582-5p<br>hsa-miR-20a-3p<br>hsa-miR-374a-5p<br>hsa-miR-20a-5p<br>hsa-miR-126-5p<br>hsa-miR-200b-5p<br>hsa-miR-144-5p<br>hsa-miR-203a<br>hsa-miR-210-3p	
Motedayyen H. (2015)	Gingival tissue	20	10	Chronic periodontitis	N/D		N/D	N/D	N/D	N/D	N/D	N/D	N/D	has-miR-146a	(31)
Stoecklin-Wasmer C. (2012)	Gingival tissue	158	40	Non-specificied	Microarray Agilent Platform	N/D	N/D	yes	N/D	N/D	N/D	N/D	N/D	hsa-miR-451<br>hsa-miR-223<br>hsa-miR-486-5p<br>hsa-miR-1246<br>hsa-miR-1260<br>hsa-miR-141	(32)
Yu-feng Xie (2011)	Gingival tissue	10	10	Chronic periodontitis	N/D	N/D	N/D	Yes	N/D	Trizol reagent (Invitrogen, CA, USA)	N/D	N/D	N/D	hsa-miR-146a<br>hsa-miR-146b<br>hsa-miR-155	(33)

Characteristics of the included studies, the different study variables are described, the analysis was carried out in two phases: identification phase where data was collected as identification method of miRNAs, RNA isolation method as well as whether the validation of the same. In a second phase, the validation phase was analyzed, where information related to the processing of the samples was collected.
